# Inflammatory Molecules in Aqueous Humour and on Ocular Surface and Glaucoma Surgery Outcome

**DOI:** 10.1155/2010/939602

**Published:** 2010-05-05

**Authors:** Barbara Cvenkel, Andreja Nataša Kopitar, Alojz Ihan

**Affiliations:** ^1^Eye Hospital, University Medical Centre Ljubljana, 1000 Ljubljana, Slovenia; ^2^Institute of Microbiology and Immunology, Faculty of Medicine, University of Ljubljana, 1000 Ljubljana, Slovenia

## Abstract

*Purpose*. To investigate the influence of inflammatory molecules in the aqueous humour and on the ocular surface on the outcome of glaucoma surgery. *Methods*. Thirty patients who needed antiglaucomatous surgery were included. The interleukin- (IL-) 8, IL-1*β*, IL-6, IL-10, tumour necrosis factor- (TNF-) *α*; and IL-12 were determined from aqueous humour preoperatively and the imprints of conjunctiva were analysed for expression of human leukocyte antigen- (HLA-)-DR after surgery by flow cytometry. The success of trabeculectomy was defined as intraocular pressure less than 21 mmHg without antiglaucoma medication. *Results*. Eyes with trabeculectomy failure at 3 months showed significantly higher TNF-*α* and IL-6 levels in the aqueous than eyes with successful surgery. Increased expression of HLA-DR on epithelial cells and antigen-presenting cells was not associated with the trabeculectomy outcome. *Conclusions*. Higher preoperative levels of TNF-*α* and IL-6 in aqueous humour may contribute to the development of inflammatory milieu and were associated with worse outcome of glaucoma surgery.

## 1. Introduction

Trabeculectomy is commonly performed in patients with uncontrolled glaucoma and with the use of antimetabolites and surgical technique modification is successful in the majority of patients [1]. The main reason for failure is episcleral fibrosis and thickening of the conjunctiva overlying the area of filtration. The inflammation and subsequent scarring are intense in the early postoperative period, in which most of the failures occur. The process of wound healing and tissue remodelling continues indefinitely after surgery and results in increasing failure rate with longer follow-up [2]. Risk factors which may influence the outcome of surgery include- previous surgery, long-term use of topical antiglaucoma medications, ocular inflammation, young age, ethnic origin, and postoperative suture lysis [2–4]. 

To understand the mechanism of filtering surgery failure the research has mainly focused on conjunctiva. Changes in the number of conjunctival fibroblasts and inflammatory cells were associated with increased risk of trabeculectomy failure. Long-term use of antiglaucoma drops with preservative benzalkonium chloride (BAK) induces subclinical inflammation of conjunctiva with over expression of human leukocyte antigen- (HLA-) DR on epithelial cells. Several cytokines were found to stimulate proliferation of Tenon's capsule fibroblasts, cells with a key role in scarring process, such as TGF-*β*, TNF, and IL-1 [5, 6]. In trabeculectomy, a fistula drains aqueous humour from the anterior chamber into the subconjunctival space. Also changes in the composition of this aqueous could influence the scarring process [7, 8]. 

In the prospective study we examined the effect of five cytokines in the aqueous humour on the outcome of trabeculectomy. We also investigated the superior bulbar conjunctiva overlying the area of filtration area for the expression of HLA-DR, the markers of subclinical inflammation, on conjunctival epithelial cells and CD80 positive cells (antigen-presenting cells) during follow-up to determine whether changes in the expression of these molecules are associated with the outcome of surgery.

## 2. Patients and Methods

Thirty patients (30 eyes) with uncontrolled intraocular pressure on maximal topical antiglaucoma medication who needed glaucoma surgery were included in this prospective study. The study protocol was approved by National Ethics Committee and all patients after receiving complete information signed informed consent form for inclusion in the study. 

At the beginning of surgery after local anaesthesia aqueous humour was obtained through the side incision made by 15° knife, avoiding any contact with limbal vessels and iris. Aqueous humour (0.05–0.1 mL) was drawn into a conventional tuberculin syringe and samples were stored at −70°C, until tested. Trabeculectomies were performed by a fornix-based conjunctival access and a scleral flap of 4 × 3 mm. In all patients mitomycin C (0.3 mg/mL) was topically applied for 3 minutes on a sponge beneath the conjunctiva. The sclerectomy was made by Kelly's punch using 2–3 bites to create an opening. Scleral flap was closed with 2–4 sutures, of those 1–2 releasable. At the end of surgery, steroid and antibiotic injections were given inferiorly. Topical steroids and antibiotics were prescribed, and after 1 month only steroid eye drops were instilled and stopped 3 months after surgery. 

Patients were scheduled for control visits 1 week, 1 month, 2, 3, 6, and 12 months after surgery, and more often if deemed necessary. 

The collection of conjunctival cells was performed by the imprinting of the ocular surface onto Millipore filter paper (versus 0.22 *μ*m pore size) after topical anaesthesia with 1-2 drops of oxybupropacaine [9]. Strips of filter paper approximately 3 × 6 mm were pressed firmly with a glass rod on the ocular surface for 5 seconds and peeled off with a forceps and put into 1 mL of phosphate buffered saline. Three imprints were collected from superior bulbar conjunctiva 1 month and 3 months after surgery. 

Surgical success was defined as intraocular pressure (IOP) less than 21 mm Hg without antiglaucoma medication and was evaluated at month 3, 6, and month 12 follow-up visits.

### 2.1. Flow Cytometry

#### 2.1.1. Analysis of Cytokines in the Aqueous Humour

A BD Human Inflammation CBA (Cytometric Bead Array) Kit was used to quantitate IL-8, IL-1*β*, IL-6, IL-10, and TNF-*α* and IL-12p70 protein levels in aqueous humour samples (BD Bioscience Pharmingen San Diego, CA, USA). Sample processing and data analysis were performed according to the manufacturer's instructions. Briefly, aqueous humour samples were incubated for 3 hours, light protected at room temperature with the six cytokine capture beads and PE-conjugated detection antibodies. After incubation samples were washed and sample data were acquired using a FACSCanto flow cytometer (BD Bioscience, San Diego CD, USA). Sample results were generated using graphical and tabular format using the BD FCAP array software (BD Bioscience, San Diego, CA, USA). 

#### 2.1.2. Analysis of Ocular Surface Imprints

Monoclonal antibodies conjugated with fluorochromes, CD80 (FITC), Anti-Cytokeratin (FITC) and Anti-HLA-DR (PE), were purchased from BD Pharmingen (San Jose, CA, USA). A two-parameter analysis was performed to determine the expression of HLA-DR on cytokeratin positive cells (epithelial cells) and on CD80 positive cells (antigen-presenting cells). Flow cytometric analysis was performed using BD FACSCanto cytometer. At least 3000 events were collected per sample. Cells were analyzed with BD FACSDiva software. The FACS data are reported as mean fluorescence intensities (MFI) and as percentages of positive cells.

### 2.2. Statistical Analysis

For statistical analysis SPSS 15.0 software for Windows was used. Because the data were not normally distributed, median, minimum, and maximum values were calculated for the levels of cytokines and for the expression of HLA-DR by epithelial and antigen-presenting cells. Mann-Whitney *U* test was used to test the hypothesis that there is no difference in the levels of cytokines and in the expression of inflammatory markers between the eyes with successful and failed surgery. The significance of the difference in the expression of HLA-DR on epithelial and antigen-presenting cells 1 and 3 months after surgery was tested with paired sample *t*-test. *P* values less than .05 were considered significant.

## 3. Results

Aqueous humour specimens from 30 patients undergoing trabeculectomy (13 females, 17 males) with the mean age 63.6 years (standard deviation 10.3 years) were analyzed for cytokines. The levels of cytokines in all patients as well as the values regarding the type of glaucoma are summarized in Table 1. 

**Figure 1:**
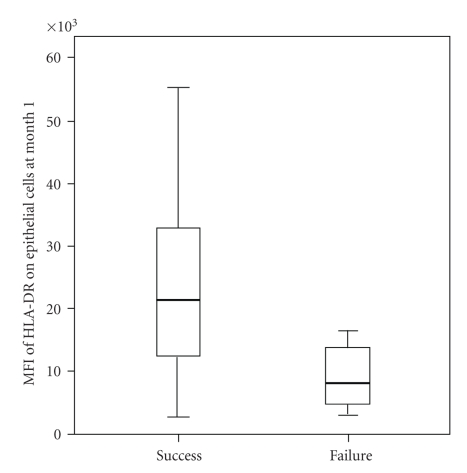
Box plot of expression of HLA-DR on conjunctival epithelial cells (MFI-mean fluorescence intensity) collected by impression cytology 1 month after surgery in eyes with surgical success and failure at 6 months (*P* = .036). The boundaries of each box indicate the 25th and 75th percentiles, the whiskers indicate the minimum and maximum values, and the line within each box indicates the median.

**Figure 2:**
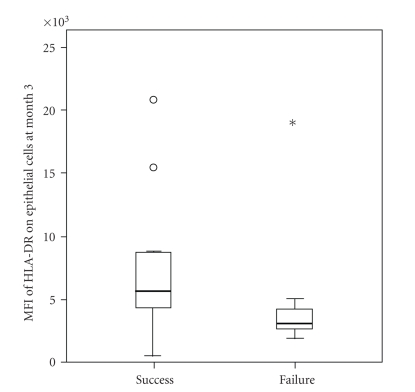
The expression of HLA-DR on conjunctival epithelial cells (MFI-mean fluorescence intensity) collected by impression cytology 3 months after surgery in eyes with surgical success and failure at 6 months of follow-up (*P* = .082). Open circles and asterisk indicate the outliers.

At 3 months of follow-up 23 eyes (patients) had successful surgery (defined as IOP less than 21 mm Hg without antiglaucoma medication), and at 6 and 12 months there were 21 eyes with successful surgery. The median values and ranges for the percentages of HLA-DR positive cytokeratin cells (epithelial cells) and CD80 positive cells (antigen-presenting cells), with their median fluorescence intensity are shown in Table 2. Mean fluorescence intensity of HLA-DR molecules on epithelial cells at 1 month and 3 months was increased in eyes with successful surgery at 12 months, but there was large scatter of the data (Figures 1 and 2).

**Figure 3:**
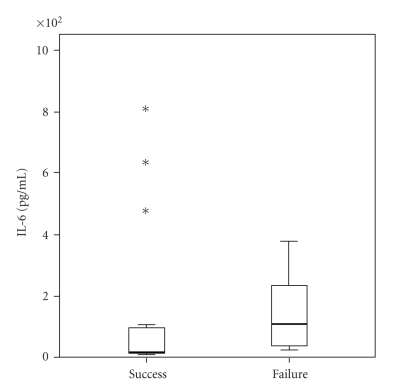
Aqueous levels of IL-6 in eyes with surgical success and failure 3 months after surgery (*P* = .031). Asterisks indicate the outliers.

**Figure 4:**
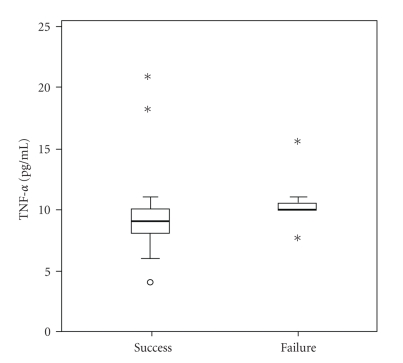
Aqueous levels of TNF-*α* in eyes with surgical success and failure 3 months after surgery (*P* = .025).

Aqueous levels of cytokines in the eyes with surgical success and failure are summarized in Table 3. We found significantly higher levels of IL-6 and TNF-*α* in eyes with surgical failure when compared to the eyes with surgical success (Figures 3 and 4). 

**Figure 5:**
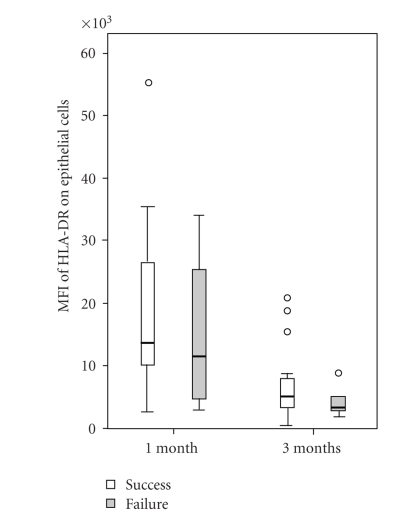
Box plot of expression of HLA-DR on conjunctival epithelial cells 1 month and 3 months after surgery in eyes with successful and failed trabeculectomy (*P* = .001).

The expression of HLA-DR on epithelial cells was significantly lower 3 months compared to 1 month after surgery (paired sample *t*-test; *P* = .001), both for the eyes with surgical success and failure (Figure 5). There was no significant difference in the percentage of HLA-DR positive epithelial and antigen-presenting cells as well as in the expression of HLA-DR by antigen-presenting cells between 1 month and 3 months of follow-up (not shown).

## 4. Discussion

Several studies have investigated conjunctival biopsy specimens from patients with glaucoma receiving long-term antiglaucoma medication and after previous ocular surgery before undergoing filtration surgery. They have shown increased number of conjunctival fibroblasts and inflammatory cells when compared to eyes without previous ocular surgery or only briefly treated-primary surgery eyes [3, 10, 11]. Such preoperative subclinical inflammation was identified as a risk factor for failure of trabeculectomy and was attributed to the upregulation of the wound healing response caused by an increase in the number and degree of activation of these inflammatory cells [3, 10]. 

Conjunctival epithelium has been studied in impression cytology specimens by flow cytometry. In eyes treated over long term without clinical inflammation, overexpression of inflammatory marker HLA-DR and IL-6, IL-8, and IL-10 was documented [11–13]. It was demonstrated that the preservative benzalkonium chloride was the major component in the eye drops responsible for toxic and immunoinflammatory effects or both in the conjunctiva [11, 14]. 

In our study, we used impression cytology specimens from the superior bulbar conjunctiva and flow cytometry to measure ocular surface inflammation 1 month and 3 months after trabeculectomy in an attempt to correlate ocular surface changes with the outcome of surgery. There was a trend of increased number of HLA-DR positive conjunctival epithelial cells collected one month after surgery in failures when compared to successes at 3-month follow-up (Table 2). In contrast to others, higher expression of HLA-DR on conjunctival epithelial cells and antigen-presenting cells was not more common in failures [15]. Interestingly, the expression of HLA-DR on epithelial cells at month 1 and 3 was higher in eyes with successful surgery than in failures, suggesting smaller ocular surface inflammation in failures (Figures 1 and 2). This cannot be explained by the difference in postoperative treatment regimen between the two groups, as all patients were receiving topical dexamethasone eye drops (Maxidex, Alcon) containing preservative benzalkonium chloride for up to 3 months after surgery that is, at the time points the specimens were collected. It was demonstrated that eyes with good intraocular pressure control have microcysts at the bleb surface containing aqueous humour, indicating a transcellular pathway of aqueous [16, 17]. Hence, the different expression of HLA-DR on epithelial cells in eyes with such functioning blebs may be also induced by the composition of aqueous.

Because the imprints of ocular surface were not collected before surgery, it remains unknown whether there was any difference in preoperative HLA-DR expression between the eyes with successful and failed surgery. In both groups there was a reduction of HLA-DR expression on epithelial cells over time, indicating diminished postoperative inflammation with longer follow-up **(** Figure 5 **)**. 

The aqueous humour is known to contain several cytokines capable of affecting fibroblast activity [18, 19]. TGF-*β* has been implicated in several ocular scarring processes including proliferative vitreoretinopathy, conjunctival wound healing, especially that occurring after trabeculectomy [20]. It has been shown to be the most potent stimulator of human Tenon's fibroblast activity. In addition, aqueous concentrations of TGF-*β*_2_ are increased in glaucomatous eyes [21]. In patients with exfoliative glaucoma previous argon laser trabeculoplasty (ALT) increased the risk of scarring because of increased aqueous levels of activated TGF-*β*_2_ compared to patients without ALT [8]. A monoclonal antibody to TGF-*β*_2_ was developed to prevent the progression of fibrosis in patients undergoing trabeculectomy, but in a randomized multicentre study the subconjunctival application of the anti TGF-*β*_2_-antibody was not different from placebo in preventing trabeculectomy failure [22].

In this study, we used flow cytometry to measure cytokines in the aqueous humour of patients undergoing trabeculectomy to evaluate whether preoperative changes in the level of cytokines could predict the outcome of surgery. The proinflammatory cytokines IL-1*β*, IL-6, IL-8, IL-12 and TNF-*α*, and IL-10, an anti-inflammatory cytokine, were analyzed preoperatively. We found that higher preoperative levels of TNF-*α* and IL-6 in the aqueous humour were present in eyes with surgical failure at 3 months (Table 3, Figures 3 and 4).

Tumour necrosis factor-*α* is synthesized by macrophages, monocytes, neutrophils, natural killer cells, and T cells [23]. TNF-*α* together with TGF-*β* is a major proinflammatory and profibrogenic cytokine expressed by ocular surface tissues. During the inflammatory process, it orchestrates the initiation of further leukocyte infiltration via adhesion molecule upregulation, dendrite cell maturation and survival, macrophage activation, and driving Th1 T cell responses in experimental autoimmune uveitis [24]. TNF-*α* together with IL-1*β* and VEGF causes breakdown of blood-retinal barrier in a murine model of experimental autoimmune uveitis [25]. Cunliffe at al. have shown that TNF and IL-1 stimulated proliferation of Tenon's capsule fibroblasts in tissue culture [6]. We propose that higher levels of TNF-*α* in aqueous humour stimulate monocyte/macrophage-chemoattractant protein-1 (MCP-1) gene and protein expression which could lead to an increase in the number of monocytes/macrophages from the subconjunctival tissue surrounding the fistula. These cells by producing proinflammatory and profibrogenic cytokines (e.g., TNF-*α* and TGF-*β*) increase activation and transformation of conjunctival fibroblasts with accelerated extracellular matrix deposition and consequently intense scarring response. Additionally, increased levels of TNF-*α* in the aqueous stimulate neovascularisation and contribute to stronger wound healing response. 

Interleukin-6 is a macrophage-derived cytokine but is also thought to be produced by ocular parenchymal cells [26]. It is generally considered to be a proinflammatory cytokine. Readily induced by TNF-*α*, IFN-*γ*, and IL-1, IL-6 promotes differentiation of B cells to plasma cells and induces synthesis of acute phase proteins, such as fibrinogen and C-reactive protein. Increased levels of IL-6 were found in aqueous humour in a murine model of experimental autoimmune uveitis demonstrating an increased local production at disease onset [27]. Also, in aqueous humour of patients with neovascular glaucoma, increased concentration of IL-6 correlated with the severity of iris neovascularisation [28]. Patients included in our study had primary open-angle and exfoliative glaucoma only. We suggest that higher levels of IL-6 in aqueous bathing the subconjuctival space after trabeculectomy may increase postoperative inflammation and lead to more intense fibrosis. 

Both TNF-*α* and IL-6 have been found elevated in aqueous humour of patients with idiopathic uveitis, Behcet's disease and Fuchs' heterochromic cyclitis [26, 29, 30]. Patients with uveitic glaucoma have also increased risk for excessive scarring after trabeculectomy surgery [31].

Interleukin-1*β*, IL-8, IL-10, and IL-12 were not found to be the risk factors for trabeculectomy failure.

The limitation of our study is a small number of patients included. Also, aqueous humour was collected at one time point only, at the beginning of trabeculectomy, and we do not know about the changes in cytokine levels during follow-up, which could support our results. Other cytokines not investigated in our study may influence the outcome of surgery as well as interactions between different cytokines, which cannot be studied in vivo. However, the study was prospective with a 12-month follow-up, a period where most of the surgical failures occur and also studied ocular surface at the site of surgery. 

The increased percentage and expression of HLA-DR on conjunctival epithelial cells and antigen-presenting cells were not associated with surgical failure. In contrast to other authors the expression of inflammatory marker HLA-DR on epithelial cells was elevated in eyes with successful trabeculectomy when compared to eyes with surgical failure [13].

## 5. Conclusions

In our study, inflammation of superior bulbar conjunctiva detected as overexpression of inflammatory molecules one and three months after surgery was not associated with subconjunctival fibrosis. Lower levels of TNF-*α* and IL-6 in aqueous humour were associated with better surgical outcome in patients undergoing trabeculectomy.

## Figures and Tables

**Table 1:** Median levels of cytokines (ranges) in the aqueous humour of 30 eyes (30 patients) and with respect to the type of glaucoma.

Cytokines (pg/mL)	All eyes (*N* = 30 eyes)	POAG (*N* = 26 eyes)	EXG (*N* = 4 eyes)	*P*
IL-8	36 (19–177)	41 (19–177)	33 (28–53)	.76
IL-1*β*	22 (15–48)	21 (15–48)	22 (18–27)	.74
IL-6	27 (9–809)	38 (9–809)	15 (14–96)	.16
IL-10	10 (3–19)	10 (3–19)	10 (9–12)	.80
TNF-*α*	9 (4–21)	9 (4–21)	8 (6–9)	.07
IL-12p70	14 (6–39)	14 (10–39)	12 (6–15)	.05

Mann-Whitney *U* test; significant at *P* < .05.

POAG: primary open-angle glaucoma.

EXG: exfoliative glaucoma.

**Table 2:** Percentage of HLA-DR positive epithelial and CD80 positive cells (antigen-presenting cells) and mean fluorescence intensity (MFI) of ocular surface inflammatory molecules obtained by impression cytology at month 1 and 3 of follow-up in eyes with successful and failed surgery.

% of HLA-DR positive cells and their MFI median values (minimum-maximum)	3 months after surgery			6 months and 12 months after surgery		
	Success (*n* = 23)	Failure (*n* = 7)	*P*	Success (*n* = 21)	Failure (*n* = 9)	*P*
% of HLA-DR positive epithelial cells at month 1	52 (11–97)	83 (65–83)	.058	58 (11–97)	52 (32–83)	.960

MFI of HLA-DR on epithelial cells at month 1	14834 (2742–55237)	16444 (3019–34050)	.906	21369 (2742–55237)	8094 (3019–16444)	**.036**

% of HLA-DR positive CD80 cells at 1 month	64.5 (26–97)	82 (70–96)	.128	65 (26–97)	68 (44–85)	.926

MFI of HLA-DR on CD80 positive cells at 1 month	2686 (592–23661)	2082 (1861–22194)	.844	2728 (592–23661)	2114 (1459–11540)	.514

% of HLA-DR positive epithelial cells at month 3	56 (7–93)	56 (30–84)	.762	55 (7–88)	69 (30–93)	.402

MFI of HLA-DR on epithelial cells at month 3	5178 (533–20735)	3450 (835–8747)	.493	6150 (533-20735)	2885 (1835–18634)	.082

% of HLA-DR positive CD80 cells at 3 month	67 (48–99)	67 (43–82)	.401	66 (48-83)	73 (43–99)	.920

MFI of HLA-DR on CD80 positive cells at 3 month	4456 (1028–17943)	6106 (1445–17808)	.595	6050 (1028–17943)	2755 (1340–17808)	.188

Mann-Whitney *U* test; significant at *P* < .05.

**Table 3:** Concentration of cytokines in aqueous humour in patients with successful and failed surgery at 3, 6, and 12 months of follow-up.

Cytokines, median (minimum-maximum) (pg/mL)	3 months after surgery			6 months and 12 months after surgery		
	Success (*n* = 23)	Failure (*n* = 7)	*P*	Success (*n* = 21)	Failure (*n* = 9)	*P*
IL-8	31 (19–177)	43 (29–70)	.478	36 (11–177)	34 (28–137)	.910
IL-1*β*	22 (15–48)	19 (17–41)	.357	22 (15–48)	21(17–41)	.504
IL-6	18 (9–809)	106 (26–375)	**.031**	20 (9–809)	75 (15–629)	.072
IL-10	10 (4–19)	11 (3–15)	.983	11 (4–19)	9 (3–15)	.341
TNF-*α*	9 (4–21)	10 (8–15)	**.025**	9 (4–21)	10 (8–15)	.101
IL-12p70	14 (6–39)	13 (11–24)	.983	14 (6–39)	13 (12–24)	.696

Mann-Whitney *U* test; significant at *P* < .05.
